# Precision oncology using a limited number of cells: optimization of whole genome amplification products for sequencing applications

**DOI:** 10.1186/s12885-017-3447-6

**Published:** 2017-07-01

**Authors:** Shonan Sho, Colin M. Court, Paul Winograd, Sangjun Lee, Shuang Hou, Thomas G. Graeber, Hsian-Rong Tseng, James S. Tomlinson

**Affiliations:** 1 Department of Surgery, University of California Los Angeles, 10833 Le Conte Ave, California, Los Angeles 90095 USA; 2 Department of Surgery, Greater Los Angeles Veteran’s Affairs Administration, 11301 Wilshire Blvd, California, Los Angeles 90073 USA; 3 Department of Molecular and Medical Pharmacology, University of California Los Angeles, 650 Charles E Young Dr S, California, Los Angeles 90095 USA; 4 UCLA Center for Pancreatic Diseases, University of California Los Angeles, 10833 Le Conte Ave., 72-215 CHS, California, Los Angeles 90095 USA

**Keywords:** Precision oncology, Whole genome amplification, Single-cell sequencing, Next generation sequencing, Multiple displacement amplification

## Abstract

**Background:**

Sequencing analysis of circulating tumor cells (CTCs) enables “liquid biopsy” to guide precision oncology strategies. However, this requires low-template whole genome amplification (WGA) that is prone to errors and biases from uneven amplifications. Currently, quality control (QC) methods for WGA products, as well as the number of CTCs needed for reliable downstream sequencing, remain poorly defined. We sought to define strategies for selecting and generating optimal WGA products from low-template input as it relates to their potential applications in precision oncology strategies.

**Methods:**

Single pancreatic cancer cells (HPAF-II) were isolated using laser microdissection. WGA was performed using multiple displacement amplification (MDA), multiple annealing and looping based amplification (MALBAC) and PicoPLEX. Quality of amplified DNA products were assessed using a multiplex/RT-qPCR based method that evaluates for 8-cancer related genes and QC-scores were assigned. We utilized this scoring system to assess the impact of de novo modifications to the WGA protocol. WGA products were subjected to Sanger sequencing, array comparative genomic hybridization (aCGH) and next generation sequencing (NGS) to evaluate their performances in respective downstream analyses providing validation of the QC-score.

**Results:**

Single-cell WGA products exhibited a significant sample-to-sample variability in amplified DNA quality as assessed by our 8-gene QC assay. Single-cell WGA products that passed the pre-analysis QC had lower amplification bias and improved aCGH/NGS performance metrics when compared to single-cell WGA products that failed the QC. Increasing the number of cellular input resulted in improved QC-scores overall, but a resultant WGA product that consistently passed the QC step required a starting cellular input of at least 20-cells. Our modified-WGA protocol effectively reduced this number, achieving reproducible high-quality WGA products from ≥5-cells as a starting template. A starting cellular input of 5 to 10-cells amplified using the modified-WGA achieved aCGH and NGS results that closely matched that of unamplified, batch genomic DNA.

**Conclusion:**

The modified-WGA protocol coupled with the 8-gene QC serve as an effective strategy to enhance the quality of low-template WGA reactions. Furthermore, a threshold number of 5–10 cells are likely needed for a reliable WGA reaction and product with high fidelity to the original starting template.

**Electronic supplementary material:**

The online version of this article (doi:10.1186/s12885-017-3447-6) contains supplementary material, which is available to authorized users.

## Background

**Fig. 1:**
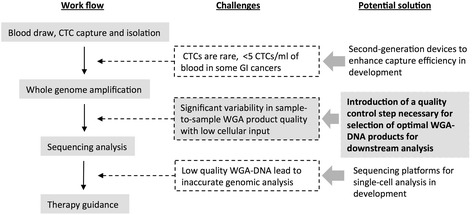
Workflow of CTC sequencing and associated challenges. Several challenges exist in CTC sequencing analysis. Quality control (QC) for the amplified DNA product is paramount, as low quality WGA products lead to failed/inaccurate downstream sequencing applications. A QC step prior to costly downstream sequencing applications helps reduce avoidable costs associated with failed sequencing

“Liquid biopsy” of circulating tumor cells (CTCs) has been suggested in many recent studies as an ideal biopsy technique for precision oncology applications [1–5]. CTCs are thought to arise from both primary and metastatic lesions, allowing for a more comprehensive representation of the tumor genomic make-up [6]. Furthermore, the need for only a simple peripheral blood draw in “liquid biopsy” makes it amenable to repeated samplings without incurring significant costs or risks to patients. Although successful CTC enrichment, capture and downstream molecular analysis has been described, major obstacles still remain prior to its clinical translation [6, 7] (Fig. 1). One major challenge is the limited number of CTCs available for molecular analysis. The number of CTCs obtainable from a single peripheral blood remains highly limited, with most studies showing <5 CTC/mL from a single peripheral blood [3, 8]. For many GI cancers, especially pancreas ductal adenocarcinoma, CTCs are even more limited [9, 10]. Thus, for molecular analysis to be performed using CTCs, the limited amount of genomic materials available from CTCs must undergo whole genome amplification (WGA) to generate adequate quantities of DNA for downstream sequencing analysis.

Despite recent advances in WGA techniques, amplification processes remain prone to uneven amplifications, resulting in amplification bias [11–14]. For heterozygous sites, this can result in a total loss of one allele, a phenomenon called allele drop out (ADO) [15, 16]. This is especially problematic when working with a small number of cells, as in CTC analysis, as the stochastic variation in the WGA process is exacerbated when starting with low copies of genomic input [17, 18]. This results in considerable variability in sample-to-sample quality when working with CTCs [15]. Low-quality WGA products with significant degrees of amplification bias and ADO are inappropriate for precision oncology applications as they fail to accurately represent the original genomic template. Therefore, one must be able to accurately differentiate between high- and low- quality WGA products in order to ensure accurate sequencing results for guiding cancer therapy.

Currently, however, quality control metrics and selection criteria for high-quality WGA products from single cells have not been sufficiently defined. Much of the existing literature utilizing whole genome amplified DNA lack analysis of the quality of WGA samples being used. Given the known sample-to-sample variability in minimal template WGA products, WGA quality must be defined in order to accurately interpret and compare WGA-DNA derived data. Furthermore, although prior studies have shown improved WGA quality with increasing amount of genomic template input [19], the number of CTCs needed to generate a WGA product suitable for sequence analysis to be utilized in precision oncology strategies remain unknown.

In the current study, we sought to define strategies for selecting and generating optimal WGA products from samples ranging from one to twenty cells as it relates to their potential applications in precision oncology strategies. To this end, we developed a quality control (QC) assay to help facilitate the selection of high quality WGA product suitable for use in downstream sequencing applications, including point mutation detection, array comparative genomic hybridization (aCGH) and next generation sequencing (NGS). In order to better understand the key determinants of WGA quality, we compared various WGA methods in addition to the number of input cells to determine their influence upon amplification reactions. We then used our findings to develop a modified multiple displacement amplification (MDA) protocol with a notable improvement in amplified DNA quality over the conventional MDA protocol. Ultimately, we utilized these findings to determine the “threshold” number of cells needed for reliable molecular analysis that could be utilized in precision oncology strategies.

## Methods

### Cell lines

Pancreatic cancer cell line HPAF-II was obtained from American Type Culture Collection (ATCC, Virginia, USA), and grown using EMEM medium (ATCC) supplemented with 10% fetal bovine serum (ATCC) and 100 U/mL penicillin-streptomycin (ATCC). All cell lines were grown at 37 °C with 5% CO_2_ and were routinely passaged at 80% confluence using an iso-osmotic sodium citrate solution for cell release (Thermo, Massachusetts, USA).

### Laser micro dissection

In preparation for laser microdissection, cells were released from the culture plates using the iso-osmotic sodium citrate solution (Thermo). Following a wash with the culture medium, each cell line was diluted to a density of 1000 cells per 100 μL. Approximately 1000 cells (100 μL) were smeared on PEN membrane slides (Leica, Wetzlar, Germany), air-dried for 10 min, and fixed with 100 μL of 100% ethanol. Cells were then isolated using the PALM MicroBeam laser microdissection system (Zeiss, Oberkochen, Germany). 1, 5, 10 or 20 cells were laser microdissected and collected into 200 μL opaque tube caps (Zeiss) using the laser pressure catapult function. Cell transfer to the tube cap was confirmed by imaging the cap prior to cap closure using the cap-check function.

### Whole genome amplification

Isolated cells were subjected to genomic DNA isolation and WGA using one of the three commercially available single-cell WGA kits according to the manufacturer’s protocol: REPLI-g Single-Cell Kit (Qiagen, California, USA), Multiple Annealing and Looping Based Amplification Cycles (MALBAC) Single-cell WGA Kit (Yikon Genomics, Beijing, China) and PicoPlex Single-Cell WGA Kit (Rubicon, Michigan). Reactions were performed a minimum of 3 times for all conditions tested. WGA products were purified using the QIAquick PCR Purification Kit (Qiagen) and quantified with NanoDrop 2000 (Thermo), in keeping with a previously described CTC molecular analysis methodology [15].

Modified MDA protocol was performed using the same reagents available from the REPLI-g Single Cell Kit (Qiagen), with modifications made in the cell lysis step and the final amplification step. Cell lysis was performed over a course of 30 min to ensure complete lysis of genomic material from isolated cells (as opposed to 10 min recommended by the manufacturer). Prior to the final amplification step, the 50-ul MDA reaction mix was mixed for 30 s by pipetting up and down and then partitioned into 16 individual reactions (approximately 3ul each) and the amplification occurred at 30 °C for 8 h. This resulted in 16 individual MDA reactions with reduced individual reaction volumes (3ul instead of 50ul), all taking place in parallel. Following the amplification step, contents within the 16-wells were collected into one tube, followed by the purification and quantification step as described above.

### Development of quality control (QC)-score for WGA-DNA

Prior literatures have described the role of multiplex PCR in evaluating DNA quality isolated from formalin-fixed paraffin embedded (FFPE) tissues prior to downstream analysis using aCGH [20]. Based on this concept, we developed an 8-gene multiplex PCR/quantitative PCR (qPCR) based QC assay for evaluation of WGA-DNA quality. We selectively chose for 8 cancer-related genes that are considered molecular targets for targeted cancer therapy and therefore highly implicated in guiding therapeutic decisions. This way, the QC assay identifies WGA products suitable for precision oncology applications by evaluating for the coverage and “accessibility” of these important genomic locations within the WGA-DNA. Genes evaluated by the QC assay included BRAF, EGFR, KIT, KRAS, NRAS, PIK3CA, PTEN and P53.

Score of 0–8 was assigned based on the number of genes successfully amplified and detected using multiplex PCR and qPCR. Failure to detect one or more of the 8-gene from the WGA product signifies lack of coverage or potential ADO during the WGA process, both of which indicate a poor quality WGA product and jeopardizes accurate representation of the original starting genome. Thus, we only gave WGA-DNA products with 8 out of 8 score a “pass” and deemed them fitting for further downstream analysis.

A secondary QC-score was generated to confirm the internal validity of the original QC-score based on the 8-cancer related genes. The secondary QC-score was generated from a distinct set of 8 primer pairs representing 8 housekeeping genes: NDUFA, UQCRC, ACTG, CYB5A, GABA-RAPL, MIF, MYC, PRPH.

### Multiplex PCR and quantitative PCR (qPCR)

WGA products were subjected to multiplex PCR pre-amplification followed by qPCR assay for detection of amplified targeted genes. Multiplex PCR was performed using primer sets representing genes mentioned above. Primer sets used for the primary QC assay included: BRAF (forward 5′ – TAC TGC TCT TTC TTC TCC AAC AC – 3′; reverse 5′ – CCT GAT TGT ATT TGA GAT CTA GTA GGG – 3′) EGFR (forward 5′-CAG CCT TCT CCG TAA TTA GCA T – 3′; reverse 5′ – TGA CAC AGA TAA TTG TCC CAC AG – 3′), KIT (forward 5′ – GGC ATT GAG GAG GGA TAG TAA AT – 3′; reverse 5′ – CTG AAC AAT TTG CTT GAA TGT TGG – 3′), KRAS (forward 5′ – GTG TTA CTT ACC TGT CTT GTC TTTG – 3′; reverse 5′ – GCC TTC TAG AAC AGT AGA CAC AA – 3′), NRAS (forward 5′ – AAT GGA ATC CCG TAA CTC TTG G – 3′; reverse 5′ – GAT GAT GTA CCT ATG GTG CTA GTG – 3′), PIK3CA (forward 5′ – AGG GCA AAT AAT AGT GGT GAT CT – 3′; reverse 5′ – CAG CAA TTA CTT GTT CTG GTA CAC – 3′), PTEN (forward 5′ – CTT TCT CTA GGT GAA GCT GTA CT – 3′; reverse 5′ – GGT TCA TTG TCA CTA ACA TCT GG – 3′) and P53 (forward 5′ – AAG AGA AGC AAG AGG CAG TAA G – 3′; reverse 5′ – CTT AGG CTC CAG AAA GGA CAA G – 3′). Primer sets used for the secondary QC assay included: NDUFA7 (forward 5′ – TGC TCT GGA TGT GAA GAT GCC A – 3′; reverse – 5′ – TTC CAG GTA AAT CCA GCC CAG G – 3′), UQCRC1 (forward 5′ – CAG CCA GTC AGC ATC ATC CAA C – 3′; reverse 5′ – GAA AGC CGG ATT GCG GTA ACA T - 3′), ACTG1 (forward 5′ - GCT CAA TGG GGT ACT TCA GGG T – 3′; reverse 5′ – GTG GAC GTT ACG TAA AAG GCC C – 3′), CYB5A (forward 5′ – GGC AAC GCT TAG ACT CTG TGT G – 3′; reverse 5′ – CTG CCC TTG GCC TAA CTA ACC T – 3′), GABARAPL2 (forward 5′ – CCA GCC AAT TCA TGA GTC GGT G – 3′; reverse 5′ – CCT GAC AAC TCG CAA GTA GCA C – 3′), MIF (forward 5′ – AGA AGT CAG GCA CGT AGC TCA G – 3′; reverse 5′ – GGC ACG TTG GTG TTT ACG ATG A – 3′), MYC (forward 5′ – GGA TAG CTC TGC AAG GGG AGA G – 3′; reverse 5′ –TCG TCG CAG TAG AAA TAC GGC T – 3′), PRPH (forward 5′ – GTT CCT CAA GAA GCT GCA CGA G – 3′; reverse 5′ – CGT TAG ACT CTG GAT CTG GCG T – 3′). Details of multiplex PCR pre-amplification is available in the Additional file 1: Supplementary Material. Briefly, PCR reactions were carried out on a C1000 Thermal Cycler (Bio-Rad) with the Multiplex PCR Plus Kit (Qiagen) using total volumes of 17 uL per reaction. The reaction conditions were as follows: 95 °C for 15 min, denaturation at 94 °C for 30 s, annealing at 64 °C for 30 s, and extension at 72 °C for 30 s for a total of 10 cycles, with a final step of 72 °C for 10 min.

Following the pre-amplification step by multiplex PCR, the resulting amplified products were analyzed and detected using qPCR. Reactions took place on BioRad CFX-96 real time system (BioRad) using the QuantiTect SYBR Green PCR Kit (Qiagen). A 25-ul reaction mixtures were prepared, which contained 12.5 ul of the SYBR Green PCR Master Mix (Qiagen), 9.5 ul of RNA-grade water, 1 ul of individual primers sets (10uM) and 2ul of the multiplex PCR product. The reaction condition were as follows: 95 °C for 15 min, followed by 35 cycles of 94 °C for 15 s, 64 °C for 20 s, and 72 °C for 20 s. The plate was read following the extension step at 72 °C. Melting curve analysis was performed between 70 and 95 °C at 0.5 °C intervals. Real-time PCR data were reviewed and analyzed using the CFX manager (BioRad). Specificity of the PCR amplification product was determined using melting curve analysis. PCR products with melting-temperature (Tm) matching the expected value based on primer sequence, and threshold cycle (Ct) <30 were counted as reliable amplification and detection.

### Array-CGH

Sample WGA-DNA and reference DNA were differentially labeled with cyanine-3 (CY3) and cyanine-5 (Cy5) dyes using the GenetiSure Amplification and Labeling Kit (Agilent) according to the manufacturer’s protocol. Briefly, a 15.5 ul reaction mixture containing sample or reference DNA, Random Primer Mix and water was placed in 98 °C for 3 min for DNA denaturation and transferred to 4 °C for 3 min. Next, 9.5 ul of Labeling Master Mix containing 5ul of 5× Reaction Buffer, 2.5ul of 10× dNTP Mix, 1.5 ul of Cy5-dUTP/Cy3-dUTP and 0.5 Exo (−) Klenow were added to each tube containing the sample/reference. DNA labeling reaction was performed in 37 °C for 45 min followed by inactivation step in 65C for 10 min. Labeled DNA was purified using the Post Labeling Purification Columns (Agilent) according to the manufacturer’s protocol. Purified labeled DNA samples were prepared for hybridization, which took place on Agilent 8x60K CGH microarray slides at 67 °C for 6 h. Following the hybridization, the slides were washed per manufacturer protocol, and prepared for scanning using the Agilent SureScan Microarray Scanner (Agilent). Microarray image was prepared and analyzed using the Agilent CytoGenomics software (Agilent).

### Sequencing library preparation and sequencing

Purified WGA products were sheared to generate DNA fragments of 350 bps using the Covaris sonicator (Covaris). Following cleanup of the sonicated DNA, end-repair and ligation were performed using the KAPA DNA Library Preparation Kit (KAPA Biosystems) according to the manufacturer’s protocol, followed by library amplification by PCR. Sequencing was performed on an Illumina HiSeq 2000 using random primers and pair-end reads of 75 bps (2X75bps).

### Sequencing analysis and visualization

The sequencing read data was analyzed using Ginkgo (http://qb.cshl.edu/ginkgo), an open-source platform for the analysis of single-cell copy-number variations (CNV). This analysis platform was developed specifically to address the unique challenges of single-cell sequencing data, and incorporates built-in computational tools for data optimization. Specifically, limitations associated with low depth of sequencing coverage, amplification bias and inflated read counts from poorly assembled genomic regions were addressed in Ginkgo’s analysis. Detailed description of the computational methodology and correction tools are provided by Garvin et al. [21]. Mapped reads were binned using variable length-binning and underwent GC bias normalization prior to segmentation using circular binary segmentation. Quality metrics data, including Lorenz curve, histogram of read count distribution and index of dispersion were obtained as a part of the Ginkgo analysis pipeline. Sequencing data visualization was performed using Nexus software (Biodiscovery, inc.).

### KRAS PCR and sanger sequencing

PCR amplification of KRAS exon 2 was performed using the following primer (Integrated DNA Technologies): Forward 5′ – AAG GTA CTG GTG GAG TAT TTG – 3′ and Reverse 5′ – GTA CTC ATG AAA ATG GTC AGA G – 3′, with expected amplicon length of 295 bps. PCR reactions were carried out on a C1000 Thermal Cycler (Bio-Rad) with Platinum PCR SuperMix High Fidelity Kit (Invitrogen) using total volumes of 50 μL per reaction according to the manufacturer’s protocol. The reaction conditions were as follows: denaturation at 94 °C for 30 s, annealing at 55 °C for 30 s, and extension at 68 °C for 45 s for a total of 40 cycles. The PCR products were purified using the QIAquick PCR Purification Kit (Qiagen) and eluted into 50 uL of nuclease-free water (Qiagen). DNA was diluted to a concentration of 10 ng/uL based on Nanodrop quantification of the PCR product. Automated dideoxy terminator sequencing was performed by capillary electrophoresis by the UCLA GenoSeq Core on an ABI 3730 DNA analyzer using Big Dye Terminator chemistry (Applied Biosystems). All sequences were analyzed by manual inspection of the individual trace files using Four Peaks (Nucleobytes).

## Results

### WGA product variability in single-cells

We applied our 8-gene QC assay to single-cell WGA-DNA samples. We tested three different WGA reaction methods: MDA, MALBAC and Picoplex. Total of 18 individual HPAF-II cells were isolated using laser micro-dissection and used in the respective WGA methods. The experiment was repeated 6 times in order to account for the variability expected with the single-cell WGA process. QC assay was performed and the QC-score was assigned for each WGA product.

**Fig. 2:**
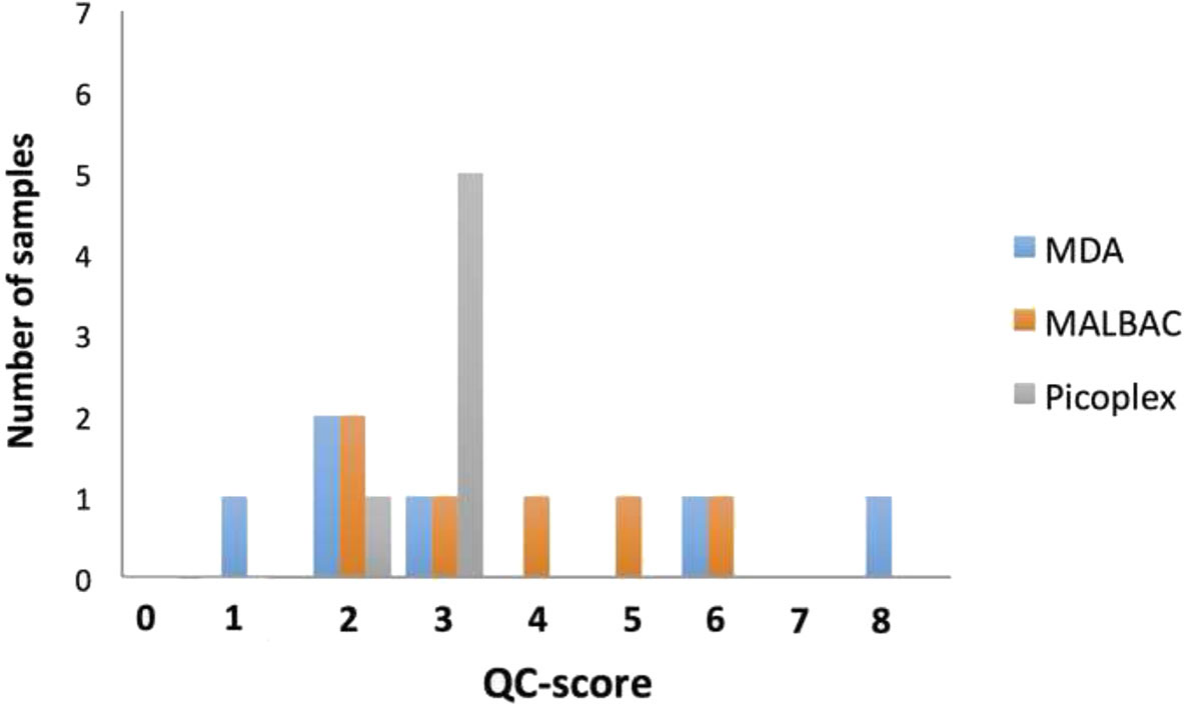
QC-scores for single-cell WGA products. Single-cell WGA was performed using MDA, MALBAC and PicoPLEX. Significant sample-to-sample variability in amplified DNA quality was noted, as measured by our 8-gene QC score

Despite using a clonally expanded cell line and performing WGA reactions in a parallel fashion under the same condition, we noted significant variability in sample-to-sample WGA-DNA quality as assessed by the 8-gene QC assay (Fig. 2). QC-scores for MDA amplified single-cells ranged between 1 and 8. Similar variability in amplified DNA quality was noted for MALBAC, with QC-scores ranging between 2 and 6. Although Picoplex resulted in less quality disparity, majority of samples achieved scores of only 3. QC-score profiles for the MDA and MALBAC methods were overall similar, but the WGA product that passed our QC criteria (QC-score = 8) was only found in the single-cell sample amplified with using the MDA method.

### Determination of number of cells needed to achieve reproducible WGA product quality

We noted significant random variations in WGA product quality when using single-cells as starting template (Fig. 2). Increasing the amount of starting genomic template has been shown to improve WGA product quality [19, 22]. Thus, we hypothesized that there would be a certain “threshold” number of cellular input above which a reliable and reproducible WGA process is possible, i.e. a number of cells which will achieve a “pass” in our QC step on a consistent basis.

**Fig. 3:**
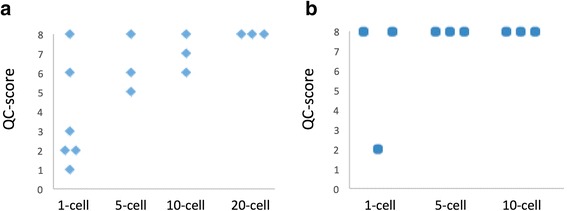
Number of cells used for genomic template input and WGA-DNA quality. **a** Conventional MDA. Overall QC-scores improve as the number of cells used for starting cellular input increases. 20-cells are required in order to attain a passing QC-score of 8 reproducibly. **b** Modified MDA**.** A passing QC**-**score of 8 is achieved reproducibly from 5-cells (as opposed to 20-cells) using the modified MDA protocol

In order to test this hypothesis, 5-cell, 10-cell and 20-cell samples of HPAF-II cells were cut and isolated using a laser micro-dissector. WGA was performed using the MDA method, followed by the QC assay and assignment of the QC-scores (Fig. 3a). Less variability in amplified DNA quality was noted when 5 or more cells were used as starting genomic template. All samples persistently achieved a QC-score of ≥5 (5-cell) or ≥6 (10-cell), as opposed to single-cell WGA reactions with scores ranging from 1 to 8. Furthermore, at least one of the triplicates in 5-cell and 10-cell group passed the QC-step (QC-score = 8). However, a highly reliable WGA process with all of the WGA products passing the QC step could not be achieved until 20 cells were used as starting genomic template input.

### Modified MDA reaction to improve quality of WGA product from limited template samples.

With the existing MDA protocol, multiple single-cell WGA reactions must be performed in order to obtain one high-quality WGA product suitable for downstream analysis (Fig. 2). Even when more than a single-cell was used, a reliable WGA reaction with its amplified DNA product passing the QC step on a consistent basis could not be achieved until 20 cells were used (Fig. 3a). For many GI cancers that are known to generate only few CTCs (i.e. pancreas ductal adenocarcinoma), obtaining 20 CTCs from a single peripheral blood draw may be unrealistic.

Given these problems, we sought to develop a modified WGA protocol that would reduce the sample-to-sample variability in DNA quality and lower the number of starting cells needed to achieve a reliable WGA process. Multiple prior reports have described the benefit of performing MDA by partitioning the reaction into parallel smaller volume reactions [16, 18, 22]. The small reaction volumes and template DNA partitioning restricts the degree of aberrant preferential amplification, leading to a more uniform WGA process overall. Based on this principle, we developed a modified MDA protocol with key changes in two aspects: (1) increasing the cell lies step from 10 min to a minimum of 30 min to ensure adequate release of genomic materials from cells, and (2) partitioning the final MDA reaction into 16 individual reactions containing ~3ul each on a 96-well plate, prior to the final isothermal amplification step at 30 °C for 8 h.

WGA products obtained using this modified MDA protocol resulted in improved reproducibility and higher QC-scores overall (Fig. 3b). Amplification reaction gain was overall lower for the modified MDA products compared to the conventional MDA, with reduction of approximately 50% on average for each sample undergoing the modified MDA protocol (Additional file 2: Table S1). For single-cells, 2 of 3 WGA reactions resulted in a product with the perfect QC-score of 8, compared to 1 in 6 WGA reactions using the conventional MDA protocol. Importantly, for 5-cell and 10-cell samples, all WGA reactions generated products that passed our QC criteria (Fig. 3b). The modified MDA protocol in our hands effectively reduced the “threshold” number of cells needed for a reliable WGA reaction down to 5 cells from 20 cells, well within the number of CTCs attainable from a single peripheral blood draw.

### WGA product QC assay using 8- vs. 16-gene multiplex PCR

Our proposed 8-gene QC assay evaluates 8 genomic locations within a WGA product. Evaluating more loci in theory provides more comprehensive evaluation of the amplified DNA product. We tested whether there was any benefit to evaluating more genetic loci beyond the 8-cancer genes during the QC process. To test for this, we performed a secondary QC assay using a different set of 8-housekeeping genes on the same single-cell conventional MDA products, as well as the 1/5/10-cell modified MDA products (Additional file 2: Table S2A and B). The original (8-cancer gene) and the secondary (8-housekeeping genes) QC assays generated highly concordant QC-scores for all of the samples tested. All of the samples that passed the QC-step based on the original QC assay also passed using the secondary QC assay. For the sole modified MDA sample with the low QC-score (sample 1-C), the secondary QC also resulted in a similarly low QC-score indicating poor quality WGA-DNA. Thus, evaluation of more genetic loci beyond the original 8-cancer genes did not provide any additional QC information and did not change QC results for any of the samples tested.

### Using 8-cancer gene QC assay to select samples for downstream aCGH and NGS applications

**Fig. 4:**
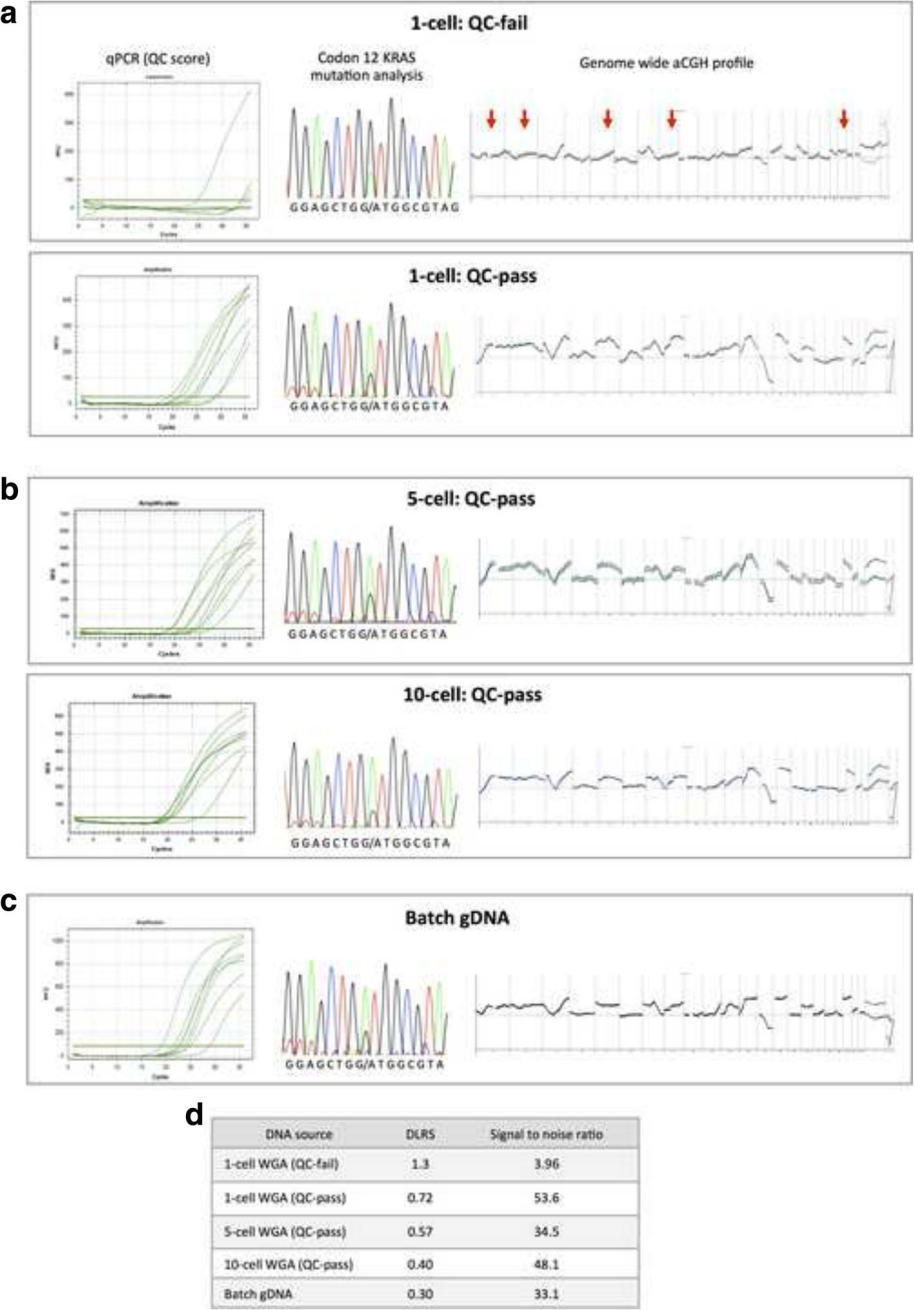
Point mutation detection and aCGH analysis using **a** single-cell WGA products that passed (QC-pass) or failed (QC-fail) the quality control step, **b**) 5 and 10-cell WGA products with passing QC, and **c** unamplified, batch genomic DNA. Single-cell WGA products (QC-fail vs. QC-pass) exhibit significantly different downstream analyses results despite using cells isolated from a clonally expanded cell line. The single-cell WGA product with the passing QC (QC-pass) generated Sanger sequencing and aCGH results that more closely resembled that of the unamplified batch gDNA compared to the single-cell WGA product failing the QC (QC-fail). Red arrows signify the areas of alteration that were detected in QC-pass and batch gDNA, but not in QC-fail. **d** aCGH quality metrics (DLRS and signal-to-noise ratio) for 1, 5 and 10-cell WGA products and batch gDNA. Improved DLRS values were associated with WGA products that passed the QC step as well as using an increasing number of starting cellular input

**Fig. 5:**
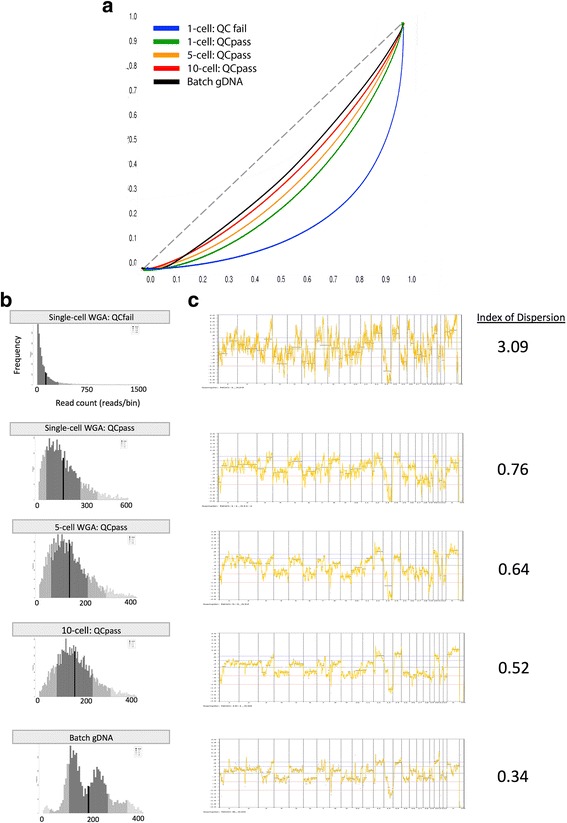
NGS application using 1, 5 and 10-cell WGA products and batch gDNA. **a** Lorenz curve illustrating the amplification bias in read coverage. Lorenz curve provides information on the uniformity of the sequencing reads distribution. Perfect coverage results in a straight line with slope of 1 (y = x) as shown by the dotted line. The wider the curve below the line of y = x, the lower the coverage uniformity and greater the amplification bias. Single-cell WGA product passing the QC-step (QC-pass) is observed to have less amplification bias compared to the single-cell WGA product that failed the QC-step (QC-fail). As the number of starting cellular input increases, the degree of amplification bias becomes even less pronounced, approaching that of unamplified batch gDNA by 10-cells. **b** Histogram of read count distribution. Wider range of distribution without a distinct peak (as seen for single-cell WGA: QC-fail) signifies worse coverage dispersion. **c** Genomic profiles generated from NGS sequencing data. Significantly less “noise” is observed with single-cell QC-pass when compared to single-cell QC-fail. Increasing the number of starting cellular input further improves the data quality, as reflected in the decreasing index of dispersion

We performed point mutation detection, aCGH and NGS using single-cell WGA products that either passed (WGA-QCpass) or failed (WGA-QCfail) the QC-step. Figures 4 and 5 illustrate the relationship between QC results and performances in various downstream applications.

Point mutation detection within the KRAS gene was successful in both QCpass and QCfail WGA products (Fig. 4a). However, while the QCpass WGA sample exhibited a mutant to wild-type allelic ratio closely matching that of the batch gDNA (HPAF-II cells are known to have 4 to 1 mutant:wild-type allelic ratio), the QCfail WGA sample exhibited evidence of amplification bias, demonstrated by an altered allelic ratio.

When we compared aCGH performances between the two single-cell WGA products, WGA-QCpass resulted in a notably better derivative log2 ratio spread (DLRS) and signal-to-noise ratio compared to WGA-QCfail (Fig. 4d). DLRS is a key quality metric for aCGH data measuring the point-to-point consistency or “noisiness” in data, with high values indicating poor signal-to-noise relationship and difficulty in assessing true copy number variation (CNV) status. The poor quality metrics (DLRS: 1.3, signal-to-noise ratio: 3.9) associated with WGA-QCfail renders its aCGH data unfit for meaningful analysis and interpretation. On the other hand, DLRS of 0.72 and signal-to-noise ratio of 53.6 associated with WGA-QCpass meets the quality threshold for single-cell derived aCGH set forth by Agilent Technologies [23]. Gain and loss profiles generated from aCGH data are as shown in Fig. 4c. Comparison between WGA-QCpass and WGA-QCfail reveals disparities in single-cell aCGH profiles, even though clonally expanded HPAF-II cells were used for both. When compared to the unamplified batch gDNA, we found multiple areas of alterations that were detected with WGA-QCpass, but not with WGA-QCfail (Fig. 4a, red arrows).

The same two single-cell WGA products were also analyzed by massive multiplex short read sequencing. Figure 5 shows the quality metrics of sequencing data associated with each WGA-DNA sample and unamplified batch gDNA. When we compare the two single-cell WGA products (WGA-QCpass and WGA-QCfail), Lorenz curves, histograms of read count frequency and indexes of dispersion all indicated a superior NGS data quality with higher coverage uniformity for WGA-QCpass compared to WGA-QCfail. The Lorenz curve provides information on the uniformity of the sequencing reads distribution. Perfect coverage results in a straight line with slope of 1 (y = x). The wider the curve below the line of y = x, the lower the coverage uniformity and greater the amplification bias. As can be seen in Fig. 5a, WGA-QCpass resulted in more uniform distribution of read depth compared to WGA-QCfail. The histogram of read count frequency also provides information on coverage dispersion (Fig. 5b). Histograms with a wide range of distribution, as seen in the sequencing data obtained using WGA-QCfail, indicates a greater degree of amplification bias. Genomic profiles generated from NGS sequencing data are as shown in Fig. 5c. Comparison between WGA-QCpass and WGA-QCfail reveals significantly more “noise” in the genomic profile derived from the latter, with a greater index of dispersion compared to that derived from WGA-QCpass.

### Determination of threshold number of cells needed for optimal performances in aCGH and NGS analysis

Although the single-cell WGA product passing our QC criteria (WGA-QCpass) performed well in point mutation detection, aCGH and NGS, room for improvement still existed when compared to the performance metrics of unamplified batch gDNA (Figs. 4 and 5). Increasing the number of starting cells has been shown to improve WGA product quality [15, 19, 22]. However, the number of cells needed for generating a WGA product that faithfully represents the unamplified, original DNA in downstream molecular analysis remains to be defined.

Using our modified MDA protocol, we lowered the numbers of cells needed to reliably pass our QC-step down to 5 cells from 20 cells. However, it remained to be answered how closely 5-cell and 10-cell WGA products approximated the unamplified DNA in aCGH and NGS analysis, and whether obtaining 5 to 10 CTCs from a single liquid biopsy is truly sufficient for sequencing applications within precision oncology strategies. Figure 4b and 5 illustrate the performances of 5-cell and 10-cell modified MDA products (QC score = 8 for both) in aCGH and NGS. As the number of cells used for starting genomic template increased from 1 to 5 to 10 cells, progressive improvement in all quality metrics of aCGH and NGS were noted. Notably, the Lorenz curves for WGA-DNA and unamplified batch gDNA nearly overlapped by 10-cells (Fig. 5a). Furthermore, by 10-cells, WGA-DNA derived aCGH and NGS genomic profiles appeared highly concordant compared to that of unamplified batch gDNA, with progressive reduction in signal “noise” and index of dispersion as the number of cells used increased (Figs. 4b and 5c). Lastly, the resolution limits of 1, 5 and 10-cell amplified products applied to the NGS analysis were assessed. By evaluating for the accuracy of CNV calling at various bin sizes (250 kb, 500 kb, 1 Mb and 2.5 Mb), we found 5 and 10-cells to require a minimum bin size of approximately 250 kb to reproduce the batch DNA result. For single-cell, bin size of approximately 2.5 Mb was needed (Additional file 3: Fig. S1). In summary, using 5–10 cells as the starting template for our modified MDA reaction generated WGA-DNA that closely approximated the unamplified gDNA in both aCGH and NGS performances.

## Discussion

CTC analysis offers unprecedented potentials for furthering precision oncology. Realization of a “liquid biopsy” through CTC sequencing helps avoid invasive and costly traditional biopsy procedures. Moreover, it allows for a dynamic monitoring of evolution in tumor genome in response to cancer therapy [24]. However, most downstream applications (i.e. aCGH, NGS) necessitate a whole genome amplification (WGA) step prior to the analysis of single/few-cell, which is known to introduce errors and biases [11–14, 25]. In the current study, we described our strategic approach for selecting and generating optimal WGA products for analysis using aCGH and NGS. We presented and validated a quality control assay for WGA product quality, and introduced a modified MDA protocol that helped improve the reproducibility and reliability of the existing WGA process. Finally, we showed that by combining our QC-criteria, modified MDA protocol and using as little as 5–10 cells as a starting template, a WGA product with high fidelity to the unamplified template DNA could be obtained.

Multiple groups have published on performance comparison of different WGA methods over the recent years, with significant differences in results and conclusions existing between these studies [14, 16, 26]. However, the majority of these studies lack analysis of the quality of WGA samples being used. Given the known high sample-to-sample variability in single-cell WGA quality, no meaningful interpretation and comparison of WGA-DNA derived data can be made without a well defined quality standard. Our proposed 8-cancer gene QC assay has the potential to fulfill this gap, as it successfully predicts the performances of WGA-DNA in downstream analysis by aCGH and NGS, in addition to providing limited sequencing information.

Prior reports have described multiplex PCR based QC assay for formalin-fixed paraffin embedded (FFPE) DNA and *Ampli1* WGA-DNA [20, 27]. The *Ampli1* WGA is a PCR-based amplification process using non-random primers, which is fundamentally dissimilar to the MDA method of non-PCR based isothermal amplification process using random primers. To date, a QC assay specific to the MDA method remains to be defined. Furthermore, no study to date has described a QC assay designed specifically to evaluate for genes implicated in cancer therapy. The eight genes included in our QC assay are all considered important molecular targets for cancer therapy, and under active investigation in the National Cancer Institute – Molecular Analysis for Therapy CHoice (NCI-MATCH) trial [28–31]. Thus, evaluation for WGA-DNA quality using this QC assay helps identify samples suitable for potential use in precision oncology strategies.

Along with the 8-cancer gene QC-assay, we have also developed a modified MDA protocol in order to help reduce the number of cells needed for a reliable WGA reaction. Our modified MDA protocol based on the principle of small volume MDA required only a 96-well plate and negated the need for labor-intensive protocols and costly special equipment described in previous works. Although this meant that we could not achieve the nanoliter reaction volumes described in prior studies, we still observed a meaningful improvement in sample-to-sample reproducibility and overall improvement in our defined QC-scores. Importantly, we noted reduced amplification reaction gains associated with our modified MDA protocol, consistent with observations made in prior literatures on small volume MDA [18, 22]. As previously described, volume restriction limits amplification reaction and decreases the overall reaction gain. However, this process can also restrict the degree of aberrant amplification of certain preferred sequences, resulting in a more uniform product overall. Although an excessively low DNA yield may signify either a poor starting DNA quality or an ineffective amplification process, an exceedingly high amplification gain is also associated with an increased amplification bias [22]. Particularly in MDA, reaction gain >10^7^ has been shown to correlate with poor amplification quality [16]. These findings suggest that the degree of amplification gain, or DNA yield, is likely an important parameter of amplified DNA quality.

In addition to improving the WGA process itself, bioinformatic computational tools are also available to improve the quality of sequencing data. A number of algorithms have been described to date, each with established efficacy in optimizing the sequencing data from single/few-cell amplified DNA [21, 32–35]. We utilized the Ginkgo (http://qb.cshl.edu/ginkgo) platform in our current analysis, which incorporates built-in algorithms designed specifically to address the challenges associated with single/few-cell amplified DNA [21]. Notably, despite the data optimization achieved by the Ginkgo algorithm, the failed QC sample remained highly biased and was unsuitable for accurate interpretation. Although statistical correction algorithms remain highly useful in single/few-cell amplified DNA analysis, generation and selection of optimal amplified DNA likely remains a key factor in successful downstream sequencing analysis.

Lastly, by combining our QC-criteria and the modified MDA protocol, we found that approximately 5–10 cells were needed to generate amplified DNA with aCGH and NGS performance approximating that of batch gDNA. Our current finding is in concordance with prior reports suggesting that 5–10 CTCs are likely needed to achieve reliable point mutation detections and aCGH analysis [15, 27]. As the interest for “liquid biopsy” and CTC analysis grows, 5–10 cells may be viewed as the minimal number of CTCs needed for reliable molecular analyses and applications in precision oncology strategies.

We acknowledge several limitations to this study. First, our described workflow employs laser capture microdissection for cell isolation, in keeping with our previously reported methodology for CTC isolation [15]. However, it is important to note that a variety of other established methods for single/few-cell isolation currently exist, including flow cytometry, micromanipulation, limiting dilutions and microfluidic/chip devise [36]. Additionally, a multitude of WGA methods are currently available and not all could be examined in the current analysis. Notably, GenomePlexVR Single-cell Whole Genome Amplification kit (Sigma-Aldrich, St. Louis, MO), Illustra Single Cell GenomiPhi DNA amplification kit (GE Healthcare, Chicago, IL) and GenoMatrix Whole Genome Amplification kits (Active Motif, Carlsbad, CA) are some of the commonly employed methods not evaluated in the current study. Application of our proposed QC technique to these other WGA methodologies may be explored. Lastly, our proposed workflow strategy remains to be evaluated in its entirety using a patient derived sample. The efficacy of several components of our current workflow, including laser capture microdissection and multiple displacement amplification, have been evaluated in patient-derived CTCs in our recent report [15]. This study has revealed that the most significant source of error in CTC sequencing analysis resulted from the variability in WGA quality, which has motivated us to develop a QC for WGA-DNA, as well as to define the threshold number of cells needed for a reliable amplification process. Although we have found these measures to potentially minimize errors in various downstream sequencing applications using a cell line, future analysis should explore their applications in patient-derived CTCs, as well as in other disseminated tumor cells.

## Conclusions

In conclusion, we presented a strategic workflow for obtaining and selecting optimal WGA products for aCGH and NGS from minimal template samples. We showed the utility of our 8-cancer gene QC assay in selecting high-quality WGA samples suitable for further molecular analysis, and demonstrated ways to improve the reproducibility and overall quality of MDA reactions. Lastly, we showed that a threshold number of 5–10 cells are likely needed to reliably and accurately represent of the original template genome using aCGH and NGS. These findings contribute to the much needed quality control criteria for minimal template WGA reactions as we explore the potential of CTCs to provide tumor specific sequence data for precision oncology strategies.

## Additional files

Additional file 1: Supplemental Method. Supplemental method on multiplex PCR. (JPEG 1392 kb)

Additional file 2: Table S1. Amplification gain of conventional vs. modified MDA reactions. **Table S2.** 8-cancer gene QC-score vs. 8-housekeeping gene QC-score. (DOCX 104 kb)

Additional file 3: Figure S1. Copy number analysis from the massive parallel sequencing data using 1-cell, 5-cell, 10-cell and batch samples. A. bin size at 250 kb, B. bin size at 500 kb, C. bin size at 1 Mb and D. bin size at 2.5 Mb. Resolution of copy-number analysis using 1, 5 and 10 cells. (DOCX 16 kb)

## Abbreviations

CNV: Copy number variation
CTC: Circulating tumor cell
MALBAC: Multiple annealing and looping based amplification cycle
MDA: Multiple displacement amplification
NGS: Next generation sequencing
QC: Quality control
WGA: Whole genome amplification
