# Supplementary figures and images for: Precision oncology using a limited number of cells: optimization of whole genome amplification products for sequencing applications
Source: BMC Cancer. 2017 Jul 1;17:457. doi: 10.1186/s12885-017-3447-6 (PMC5493892; doi:10.1186/s12885-017-3447-6)

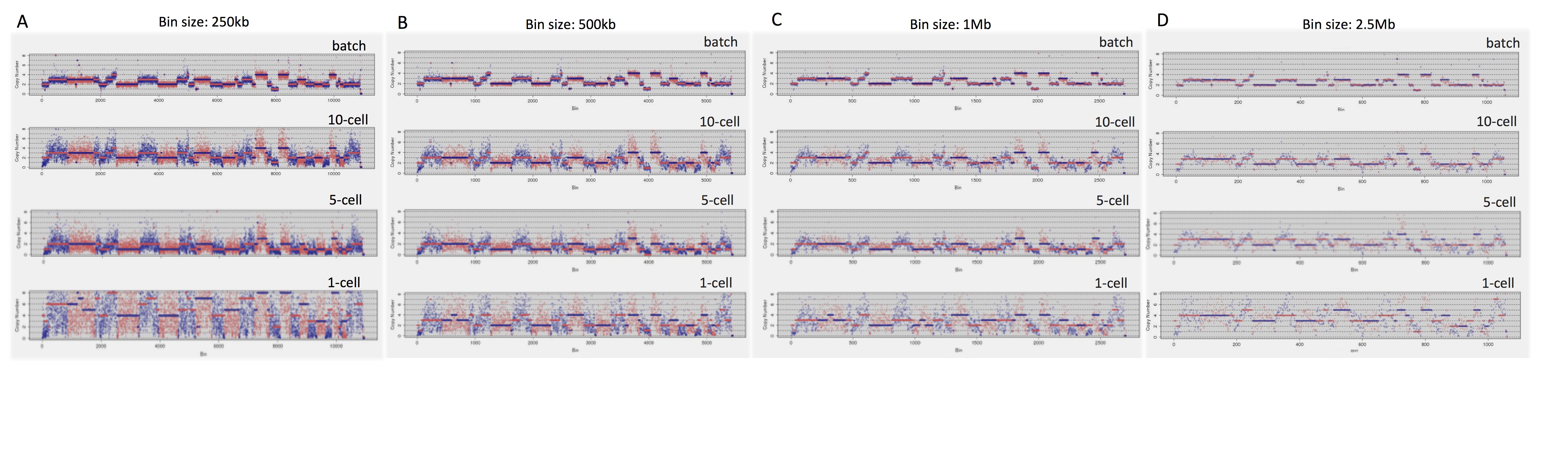

Supplement: Supplementary file 1 — Supplemental Method. Supplemental method on multiplex PCR. (JPEG 1392 kb) [file 12885_2017_3447_MOESM3_ESM.jpg]
