# Supplementary material for: Precision oncology using a limited number of cells: optimization of whole genome amplification products for sequencing applications
Source: BMC Cancer. 2017 Jul 1;17:457. doi: 10.1186/s12885-017-3447-6 (PMC5493892; doi:10.1186/s12885-017-3447-6)
Supplement: Supplementary file 2 — Amplification gain of conventional vs. modified MDA reactions. Table S2. 8-cancer gene QC-score vs. 8-housekeeping gene QC-score. (DOCX 104 kb) [file 12885_2017_3447_MOESM1_ESM.docx]

**Multiplex PCR**

Multiplex PCR was performed using the Qiagen Multiplex PCR kit. The reaction mixture contained 8.5 uL of 2x Master Mix (provides final concentration of 3mM MgCl_2_), 1.7ul of primer mix (for final concentration of 0.2uM), 100ng of template DNA and RNase-free water to bring volume to 17ul per reaction. Depending on the type of QC performed (primary vs. secondary) two different sets of 8-primer pairs were used, as described in the main manuscript. Thin-walled tubes were used for individual PCR reactions. PCR reaction was performed using the C1000 Thermal Cycler (Bio-Rad). The PCR condition was 95 ^o^C for 15min, denaturation at 94^o^C for 30 seconds, annealing at 64^o^C for 30 seconds, and extension at 72^o^C for 30 seconds for a total of 10 cycles, with a final step of 72 ^o^C for 10 min.

The quality and efficacy of multiplex PCR pre-amplification reaction was confirmed for each WGA sample undergoing the QC process. This was achieved by performing concurrent multiplex PCR reactions using a positive (batch DNA) and a negative (sterile-water) control for each individual WGA sample being tested, and subsequently evaluating their reaction outputs using a qPCR assay. For a multiplex PCR reaction to be deemed effective and reliable, all amplified genes (8-genes covered by the primer sets) should be detectable for the positive control (batch DNA) using qPCR. On the other hand, none of the 8-genes should be detected by the qPCR assay when using a negative control (sterile-water). Only those multiplex PCR reactions passing this requirement could be deemed useful for evaluating a WGA sample quality.
