# Supplementary material for: Precision oncology using a limited number of cells: optimization of whole genome amplification products for sequencing applications
Source: BMC Cancer. 2017 Jul 1;17:457. doi: 10.1186/s12885-017-3447-6 (PMC5493892; doi:10.1186/s12885-017-3447-6)
Supplement: Supplementary file 3 — Copy number analysis from the massive parallel sequencing data using 1-cell, 5-cell, 10-cell and batch samples. A. bin size at 250 kb, B. bin size at 500 kb, C. bin size at 1 Mb and D. bin size at 2.5 Mb. Resolution of copy-number analysis using 1, 5 and 10 cells. (DOCX 16 kb) [file 12885_2017_3447_MOESM2_ESM.docx]

| Conventional MDA | Amplification gain­ | Modified MDA | Amplification gain | P-value |
| --- | --- | --- | --- | --- |
| 1-cell |  | 1-cell |  |  |
| 1-a | 3.4 x10^6^ | 1-A | 1.8 x10^6^ |  |
| 1-b | 4.8 x10^6^ | 1-B | 1.8 x10^6^ |  |
| 1-c | 3.9 x10^6^ | 1-C | 3.2 x10^6^ |  |
| 1-d | 4.4 x10^6^ |  |  |  |
| 1-e | 3.9 x10^6^ |  |  |  |
| 1-f | 3.0 x10^6^ |  |  |  |
| mean ± SD | 3.9 x10^6^ | mean ± SD | 2.25 x10^6^ | **0.011** |
| 5-cell |  | 5-cell |  |  |
| 5-a | 7.4 x10^5^ | 5-A | 1.9 x10^5^ |  |
| 5-b | 8.1 x10^5^ | 5-B | 4.5 x10^5^ |  |
| 5-c | 8.5 x10^5^ | 5-C | 3.7 x10^5^ |  |
| mean ± SD | 8.0 x10^5^ | mean ± SD | 3.4 x10^5^ | **0.005** |
| 10-cell |  | 10-cell |  |  |
| 10-a | 4.6 x10^5^ | 10-A | 1.9 x10^5^ |  |
| 10-b | 3.8 x10^5^ | 10-B | 2.1 x10^5^ |  |
| 10-c | 4.2 x10^5^ | 10-C | 2.3 x10^5^ |  |
| mean ± SD | 4.2 x10^5^ | mean ± SD | 2.1 x10^5^ | **0.001** |

**Table S1**: Amplification gain of conventional vs. modified MDA reactions

**Table S2**: 8-cancer gene QC-score vs. 8-housekeeping gene QC-score

A: Conventional single-cell MDA-WGA

| Sample | 8-cancer gene QC-score | 8-housekeeping gene QC-score |
| --- | --- | --- |
| 1-a | 8 | 8 |
| 1-b | 6 | 7 |
| 1-c | 2 | 3 |
| 1-d | 3 | 1 |
| 1-e | 1 | 0 |
| 1-f | 2 | 2 |

B: Modified MDA-WGA

| Sample | 8-cancer gene QC-score | 8-housekeeping gene QC-score |
| --- | --- | --- |
| 1-A | 8 | 8 |
| 1-B | 8 | 8 |
| 1-C | 2 | 4 |
| 5-A | 8 | 8 |
| 5-B | 8 | 8 |
| 5-C | 8 | 8 |
| 10-A | 8 | 8 |
| 10-B | 8 | 8 |
| 10-C | 8 | 8 |
